# Supplementary material for: Tree species differ in plant economic spectrum traits in the tropical dry forest of Mexico
Source: PLoS One. 2023 Nov 9;18(11):e0293430. doi: 10.1371/journal.pone.0293430 (PMC10635469; doi:10.1371/journal.pone.0293430)
Supplement: S1 Table — (PDF) [file pone.0293430.s001.pdf]

Supporting information

S1 Table. Localization and general climate characteristics of the study sites. Mean values; coefficient of variation is in parentheses for annual precipitation and temperature, for rainy season precipitation, accumulated percentage, and for the maximum temperature, the month that presents this value is indicated Data was obtained from Clicom Project, National Meteorological Service, CICESE, http://clicom-mex.cicese.mx.

Site	Location	Annual precipitation (mm)	Rainy season precipitation (mm)	Annual temperature (°C)	Max. temperature (°C)
Sierra de Manantlán, Jalisco	19.2647 - 19. 4205 N; 103.5112 -104.2705 W	677.26 (26.78)	558.48 (82.55)	23.4 (3.07)	37.90 (April)
Sierra de Montenegro, Morelos	18.8939 - 18.6985 N; 99.1090 – 99.1217 W	953.96 (22.51)	844.10 (88.48)	23.0 (4.47)	40.40 (May)
Parque Nacional Huatulco, Oaxaca	15.3912 - 15.4710 N; 96.0630 - 96.1500 W	1,389.03 (33.39)	1,257.61 (90.50)	26.5 (3.6)	42.92 (April)
