# Supplementary material for: Tree species differ in plant economic spectrum traits in the tropical dry forest of Mexico
Source: PLoS One. 2023 Nov 9;18(11):e0293430. doi: 10.1371/journal.pone.0293430 (PMC10635469; doi:10.1371/journal.pone.0293430)
Supplement: S2 Table — (PDF) [file pone.0293430.s002.pdf]

Supporting information

S2 Table. Summary of the wood anatomical traits measured in six tree species of the tropical dry forest in three different sites. SD, standard deviation; Min., minimum value; Max., maximum value; CV, coefficient of variation. V_D, Vessel diameter; V^{mm-2}, vessels frequency; VI, Vulnerability Index; V_F, vessel fraction; F_D, Fiber cell diameter; F_{Dl}, fiber cell lumen diameter; F_{WT}, fiber cell wall thickness; F_F, fiber fraction; P_p, parenchyma fraction; WD, wood density. For V_D, F_D, F_{Dl}, F_{WT} values are in µm and for WD in g cm^{-3}

Species	Group I: *C. vitifolium*											
Site	Parque Nacional Huatulco				Sierra de Montenegro				Sierra de Manantlán			
Trait	Mean (SD)	Min.	Max.	CV	Mean (SD)	Min.	Max.	CV	Mean (SD)	Min.	Max.	CV
V_D	247.6 (50.9)	116.7	401.2	20.5	238.9 (44.6)	128.3	384.6	18.7	234.3 (42.4)	134.6	423.3	18.1
V^{mm-2}	1.8 (0.8)	0.4	4.5	43.3	2.5 (1.3)	0.4	6.8	52.8	3.5 (2.0)	0.4	8.1	56.0
VI	140.4 (17.2)	115.0	155.5	12.2	96.2 (12.5)	78.6	111.5	13.0	81.9 (48.5)	45.3	164.2	59.2
V_F	0.05 (0.02)	0.03	0.07	40.0	0.06 (0.02)	0.04	0.09	33.3	0.07 (0.03)	0.04	0.12	42.9
F_D	34.7 (7.0)	28.4	45.6	20.0	29.9 (6.3)	21.3	36.1	21.1	34.0 (8.7)	21.8	44.4	25.6
F_{Dl}	29.6 (9.7)	14.2	76.3	36.7	29.3 (4.6)	5.1	54.6	15.6	27.5 (13.3)	5.6	62.6	48.5
F_{WT}	2.6 (0.8)	1.5	3.4	31.3	2.4 (0.3)	1.7	2.6	12.8	2.7 (0.3)	2.2	3.0	12.4
F_F	0.29 (0.08)	0.22	0.43	27.6	0.30 (0.05)	0.34	0.46	16.7	0.30 (0.03)	0.20	0.31	10.0
P_F	0.66 (0.08)	0.56	0.77	12.1	0.66 (0.06)	0.56	0.72	9.1	0.63 (0.03)	0.57	0.65	4.76
WD	0.25 (0.04)	0.24	0.28	16.0	0.29 (0.03)	0.27	0.33	10.3	0.32 (0.03)	0.28	0.35	9.38
Species	Group I: *S. purpurea*											
Site	Parque Nacional Huatulco				Sierra de Montenegro				Sierra de Manantlán			
Trait	Mean (SD)	Min.	Max.	CV	Mean (SD)	Min.	Max.	CV	Mean (SD)	Min.	Max.	CV
V_D	190.2 (34.5)	102.7	295.9	18.1	191.1 (36.0)	66.6	276.7	18.8	172.6 (41.0)	88.1	248.6	23.8
V^{mm-2}	9.0 (2.7)	5.4	18.1	30.0	10.0 (2.9)	4.5	17.1	29.0	10.7 (3.4)	6.3	16.7	34.0
VI	21.7 (4.6)	15.9	27.0	21.2	19.7 (3.3)	14.9	23.8	16.8	17.4 (7.8)	12.0	22.9	44.8
V_F	0.20 (0.04)	0.15	0.27	20.0	0.21 (0.08)	0.13	0.32	38.1	0.18 (0.01)	0.18	0.19	5.6
F_D	23.8 (4.0)	13.2	35.3	16.8	22.7 (4.2)	11.1	36.0	18.5	34.0 (4.0)	15.1	33.3	11.8
F_{Dl}	20.0 (4.3)	8.4	31.8	20.5	18.0 (4.2)	10.9	30.7	23.3	18.3 (3.9)	5.6	29.7	21.3
F_{WT}	1.9 (0.4)	1.4	2.6	24.1	2.3 (0.4)	1.8	2.7	16.3	1.9 (0.2)	1.8	2.0	10.5
F_F	0.57 (0.08)	0.48	0.67	14.0	0.58 (0.04)	0.53	0.64	6.9	0.47 (0.11)	0.39	0.55	23.4
P_F	0.23 (0.07)	0.14	0.33	30.4	0.21(0.08)	0.10	0.28	38.1	0.34 (0.10)	0.27	0.42	29.4
WD	0.52 (0.06)	0.45	0.60	11.5	0.56 (0.15)	0.37	0.68	26.8	0.53 (0.04)	0.50	0.56	7.5
Species	Group II: *T. rosea*											
Site	Parque Nacional Huatulco				Sierra de Montenegro				Sierra de Manantlán			
Trait	Mean (SD)	Min.	Max.	CV					Mean (SD)	Min.	Max.	CV
V_D	177.5 (20.0)	131.2	238.8	11.3					177.9 (30.5)	84.6	259.9	17.1
V^{mm-2}	9.4 (2.7)	3.6	16.7	28.7					10.9 (2.1)	6.3	16.7	19.3
VI	19.1 (2.9)	16.6	22.6	15.2					16.6 (2.3)	13.2	19.0	13.9
V_F	0.16 (0.03)	0.12	0.20	18.8					0.19 (0.05)	0.13	0.23	26.3
F_D	15.0 (2.1)	10.6	23.5	14.0					14.5 (2.0)	9.7	21.6	12.9

F_{Dl}	6.2 (1.8)	2.6	13.0	29.0					7.8 (1.9)	3.3	14.0	24.4
F_{WT}	4.4 (0.8)	3.8	5.5	18.5					3.4 (0.3)	3.0	3.7	9.2
F_F	0.44 (0.08)	0.38	0.56	18.2					0.39 (0.03)	0.37	0.44	7.7
P_F	0.39 (0.04)	0.33	0.42	30.4					0.42 (0.06)	0.33	0.48	29.4
WD	0.63 (0.06)	0.54	0.67	9.5					0.53 (0.04)	0.52	0.60	7.5
Species	Group II: *P. dulce*											
Site	Parque Nacional Huatulco				Sierra de Montenegro				Sierra de Manantlán			
Trait	Mean (SD)	Min.	Max.	CV	Mean (SD)	Min.	Max.	CV	Mean (SD)	Min.	Max.	CV
V_D	184.6 (26.1)	104.8	286.8	14.1	146.1 (33.0)	72.5	245.8	22.6	159.6 (31.8)	75.7	265.4	19.9
V^{mm-2}	8.7 (1.8)	3.6	12.2	20.7	11.0 (2.5)	5.0	17.1	22.7	12.5 (6.1)	4.5	25.7	48.8
VI	21.3 (1.9)	19.4	23.1	8.9	13.6 (3.1)	10.3	25.5	22.8	15.5 (8.3)	8.3	16.6	53.5
V_F	0.18 (0.01)	0.17	0.19	5.6	0.16 (0.04)	0.11	0.20	25.0	0.19 (0.04)	0.13	0.22	21.1
F_D	14.8 (2.0)	9.4	19.7	13.5	14.1 (4.7)	7.5	26.7	33.3	14.8 (2.2)	7.6	19.5	14.9
F_{Dl}	8.8 (2.0)	4.6	14.9	22.7	7.6 (3.6)	3.1	24.9	47.4	7.0 (1.8)	1.7	11.8	25.7
F_{WT}	3.0 (0.5)	2.4	3.3	17.0	3.2 (0.8)	2.4	4.3	24.5	3.1 (0.3)	2.9	3.5	8.4
F_F	0.38 (0.05)	0.34	0.44	13.2	0.45 (0.04)	0.41	0.49	8.9	0.44 (0.04)	0.39	0.48	9.1
P_F	0.44 (0.22)	0.37	0.49	50.0	0.39 (0.02)	0.37	0.41	5.1	0.37 (0.04)	0.31	0.40	10.8
WD	0.58 (0.12)	0.48	0.75	20.7	0.73 (0.04)	0.69	0.79	5.5	0.73 (0.09)	0.62	0.87	12.3
Species	Group III: *H. brasiletto*											
Site	Parque Nacional Huatulco				Sierra de Montenegro				Sierra de Manantlán			
Trait					Mean (SD)	Min.	Max.	CV	Mean (SD)	Min.	Max.	CV
V_D					168 (38.6)	79.3	235.7	23.0	130.4(22.6)	65.8	198.3	17.3
V^{mm-2}					11.6 (2.1)	3.6	16.7	19.8	19.8 (4.7)	6.3	16.7	23.7
VI					16.6 (3.0)	11.6	19.2	18.1	6.8 (0.8)	6.2	8.0	11.8
V_F					0.18 (0.03)	0.15	0.22	16.7	0.23 (0.06)	0.15	0.30	26.1
F_D					12.6 (2.7)	6.6	20.3	21.4	13.2 (2.6)	8.5	20.2	19.7
F_{Dl}					7.0 (2.4)	2.1	12.7	34.3	4.3 (1.6)	1.4	10.1	37.2
F_{WT}					3.8 (0.1)	2.9	4.8	3.7	4.3 (0.7)	3.8	5.5	15.6
F_F					0.46 (0.05)	0.36	0.50	10.9	0.46 (0.08)	0.35	0.57	17.4
P_p					0.36 (0.04)	0.31	0.41	11.1	0.31 (0.03)	0.28	0.35	29.7
WD					0.84 (0.04)	0.77	0.87	4.8	0.93 (0.04)	0.88	0.97	4.3
Species	Group III: *L. divaricatum*											
Site	Parque Nacional Huatulco				Sierra de Montenegro				Sierra de Manantlán			
Trait	Mean (SD)	Min.	Max.	CV	Mean (SD)	Min.	Max.	CV	Mean (SD)	Min.	Max.	CV
V_D	155.4 (31.4)	66.3	263.1	20.2	143.5 (38.6)	72.4	236.8	26.9	139.2 (22.6)	41.8	228.6	16.3
V^{mm-2}	23.1 (7.2)	9.5	34.3	31.2	10.8 (1.9)	6.8	16.7	17.8	9.5 (3.6)	2.7	15.3	47.4
VI	5.9 (36.8)	3.7	9.5	21.2	13.4 (3.3)	11.2	14.9	14.2	20.7 (7.8)	12.3	31.5	34.1
V_F	0.26 (0.03)	0.22	0.30	13.0	0.17 (0.05)	0.15	0.19	11.8	0.11 (0.02)	0.04	0.18	44.5
F_D	12.1 (1.5)	8.8	17.3	12.6	12.7 (1.6)	8.5	17.1	12.6	13.3 (1.7)	8.8	18.6	12.8
F_{Dl}	4.9 (1.2)	2.8	11.9	24.5	4.5 (1.2)	1.7	8.8	26.7	5.6 (1.7)	1.9	9.8	30.4
F_{WT}	3.7 (0.2)	3.3	3.8	5.1	4.1 (0.04)	4.1	4.2	0.8	3.9 (0.6)	3.2	4.3	15.5

F_F	0.50 (0.08)	0.45	0.53	6.8	0.54 (0.04)	0.49	0.57	6.3	0.61 (0.11)	0.49	0.69	12.6
P_F	0.27 (0.07)	0.17	0.31	11.1	0.29 (0.08)	0.10	0.33	13.8	0.27 (0.10)	0.27	0.33	11.1
WD	0.91 (0.06)	0.88	0.95	3.3	0.90 (0.15)	0.26	1.00	10.0	0.94 (0.04)	0.21	1.00	6.4
