# Supplementary material for: Tree species differ in plant economic spectrum traits in the tropical dry forest of Mexico
Source: PLoS One. 2023 Nov 9;18(11):e0293430. doi: 10.1371/journal.pone.0293430 (PMC10635469; doi:10.1371/journal.pone.0293430)
Supplement: S3 Table — (PDF) [file pone.0293430.s003.pdf]

Supporting information

S3 Table. Model comparison through the akaike information criterion (AIC) and the likelihood ratio test (LRT) with species and site as a factors. For LTR, the loglikelihood value is presented and the significance level is indicated, ***, 0.001; **, 0.01; *, 0.05. V_D, Vessel diameter; V^{mm-2}, vessels frequency; F_D, Fiber cell diameter; F_{Dl}, fiber cell lumen diameter; F_{WT}, fiber cell wall thickness.

Model	Method	V_D	V^{mm-2}	F_D	F_{Dl}	F_{WT}
Species*site	AIC	40987	4724	20681	20224	6388
	LRT	20475***	-2344***	-10322***	-10094***	-3176***
Species + site	AIC	41034	4790	20711	20245	6409
	LRT	-20511	-2385	-10345**	-10112	-3194
Species	AIC	41043	4792	20718	20249	6407
	LRT	-20514***	-2388***	-10351***	-10117***	-3195***
Site	AIC	41099	4835	20797	20347	6459
	LRT	-20545	-2412	-10394	-10169	-3225
Null	AIC	41099	4832	20798	20347	6459
	LRT	-20547	-2413	-10396	-10171	-3227
