# Supplementary material for: Tree species differ in plant economic spectrum traits in the tropical dry forest of Mexico
Source: PLoS One. 2023 Nov 9;18(11):e0293430. doi: 10.1371/journal.pone.0293430 (PMC10635469; doi:10.1371/journal.pone.0293430)
Supplement: S4 Table — (PDF) [file pone.0293430.s004.pdf]

Supporting information

S4. Table. Fixed effect coefficients predicted by the linear mixed models for the wood anatomical traits in six tree species and three different sites. *C. vitifolium* and Parque Nacional Huatulco were reference categories for all models. HB, *H. brasiletto*; LD, *L. divaricatum*; PD, *P. dulce*; SP, *S. purpurea*; TR, *T. rosea*. SMA, Sierra de Manantlán; SMO, Sierra de Montenegro.

	Vessel diameter (μm)	Vessel frequency (mm^{-2})	Fiber diameter (μm)	Fiber cell lumen diameter (μm)	Fiber cell wall thickness (μm)
No. Species	6	6	6	6	6
No. tree sampled	5	5	5	5	5
No. observations	4148	966	3685	3685	3685
Intercept	241.085	1.78804	34.7326	29.4945	2.61845
Species HB	6.391	7.98366	-7.2184	-8.8503	0.77911
Species LD	-115.716	21.30140	-22.5775	-24.5379	0.98081
Species PD	-63.865	6.91322	-19.9268	-20.6870	0.38063
Species SP	-50.943	7.23072	-10.8653	-9.4390	-0.71257
Species TR	-63.588	7.65760	-19.7143	-23.2228	1.75474
Site SMA	-6.821	1.71381	-2.2693	-2.1406	-0.06480
Site SMO	-74.641	0.76500	-14.8798	-13.6562	-0.57430
HB × SMA	-110.246	8.23893	-12.0876	-14.1544	1.07135
LD × SMA	20.662	-17.23083	3.4129	2.7611	0.32630
PD × SMA	-13.506	2.05682	0.6362	0.3440	0.14664
SP × SMA	-11.791	-0.08987	0.3786	0.2623	0.05813
TR × MA	7.244	-0.23113	1.7917	3.6590	-0.93298
LD × SMO	92.807	-13.04231	15.4051	13.1931	1.06844
PD × SMO	43.462	1.54598	14.0705	12.4548	0.77020
SP × SMO	75.581	0.21639	13.6634	11.5933	0.99748
