# Supplementary material for: Tree species differ in plant economic spectrum traits in the tropical dry forest of Mexico
Source: PLoS One. 2023 Nov 9;18(11):e0293430. doi: 10.1371/journal.pone.0293430 (PMC10635469; doi:10.1371/journal.pone.0293430)
Supplement: S5 Table — (PDF) [file pone.0293430.s005.pdf]

Supporting information

S5. Table. Multiple pairwise comparison in estimated marginal mean result from linear mixed models. Estimated differences are presented and significance level. *, <0.05; **<0.01; ***, <0.001. PNH, Parque Nacional Huatulco; SMA, Sierra de Manantlán; SMO, Sierra de Montenegro; CV, *C. vitifolium*; HB, *H. brasiletto*; LD, *L. divaricatum*; PD, *P. dulce*; SP, *S. purpurea*; TR, *T. rosea*.

	Vessel diameter	Vessel frequency	Fiber cell diameter	Fiber cell lumen diameter	Fiber cell wall thickness
PNH-SMA	14.70	-0.315	3.732	4.371	-0.320
PNH-SMO	18.68	1.534	4.466	4.145	0.153
SMA-SMO	3.97	1.849	0.735	-0.226	0.474
CV-HB	62.36***	-12.515***	15.866***	18.331***	-1.2192***
CV-LD	77.9***	-11.212***	16.051***	18.991***	-1.4564***
CV-PD	53.9***	-8.274***	14.896***	16.345***	-0.7112*
CV-SP	29.7*	-7.088**	5.717**	5.117*	0.3135
CV-TR	36.21**	-7.656**	14.009***	16.884***	-1.4238***
HB-LD	15.58	1.303	0.185	0.659	-0.2372
HB-PD	-7.26	4.241	-0.970	-1.986	0.5080
HB-SP	-35.88**	5.427*	10.149***	-3.214	1.5327***
HB-TR	-26.16	4.859	-1.857	-1.447	-0.2046
LD-PD	-22.84	2.938	-1.155	-2.645	0.7452
LD-SP	-51.46***	4.124	-10.334***	-13.874***	1.7700***
LD-TR	-41.74**	3.556	-2.041	-2.107	0.0327
PD-SP	-28.62*	1.186	-9.179***	-11.229***	1.0247***
PD-TR	-18.89	0.618	-0.887	0.538	-0.7126
SP-TR	9.72	-0.568	8.292	11.767***	-1.7373***
