# Supplementary material for: Tree species differ in plant economic spectrum traits in the tropical dry forest of Mexico
Source: PLoS One. 2023 Nov 9;18(11):e0293430. doi: 10.1371/journal.pone.0293430 (PMC10635469; doi:10.1371/journal.pone.0293430)
Supplement: S6 Table — (PDF) [file pone.0293430.s006.pdf]

Supporting information

S6 Table. F values for each trait studied as a result of the ANOVA. The p value is presented in parentheses. VI, vulnerability index; V_F, vessel fraction; F_F, fiber fraction; P_p, parenchyma fraction; WD, wood density.

Model	VI	V_F	F_F	P_F	WD
Species	94.6 (<0.001)	24.9 (<0.001)	41.2 (<0.001)	105.2 (<0.001)	157.6 (<0.001)
Site	4.782 (0.012)	2.315 (0.108)	0.904 (0.41)	0.154 (0.86)	3.072 (0.059)
Species*Site	5.0 (<0.001)	5.4 (<0.001)	2.7 (0.01)	2.3 (0.03)	2.7 (0.01)
