# Supplementary material for: Tree species differ in plant economic spectrum traits in the tropical dry forest of Mexico
Source: PLoS One. 2023 Nov 9;18(11):e0293430. doi: 10.1371/journal.pone.0293430 (PMC10635469; doi:10.1371/journal.pone.0293430)
Supplement: S7 Table — (PDF) [file pone.0293430.s007.pdf]

Supporting information

S7 Table. Pearson correlation matrix between wood anatomical traits. V_D, Vessel diameter; V^{mm-2}, vessels frequency; VI, vulnerability index; V_F, vessel fraction; F_D, Fiber cell diameter; F_{Dl}, fiber cell lumen diameter; F_{WT}, fiber cell wall thickness; F_F, fiber fraction; P_p, parenchyma fraction; WD, wood density; T_H, tree height. All the correlations were statistically significant (p<0.001), except for F_{WT}-P_F; T_H - V_D; T_H - Vmm^{-2}; T_H - F_F and T_H - P_F.

	V_D	Vmm^{-2}	VI	V_F	F_D	F_{Dl}	F_{WT}	F_R	F_F	P_F	WD	T_H
V_D												
Vmm^{-2}	-0.77											
VI	0.86	-0.98										
V_F	-0.55	0.90	-0.85									
F_D	0.78	-0.78	0.83	-0.66								
F_{Dl}	0.83	-0.75	0.81	-0.60	0.96							
F_{WT}	-0.51	0.43	-0.47	0.29	-0.59	-0.76						
F_F	-0.61	0.58	-0.61	0.50	-0.49	-0.47	0.20	0.41				
P_F	0.54	-0.66	0.66	0.67	0.43	0.41	-0.13	-0.34	-0.87			
WD	-0.88	0.81	-0.86	0.67	-0.87	-0.88	0.57	0.79	0.70	-0.61		
T_H	-0.22	0.29	-0.28	0.26	-0.42	-0.46	0.45	0.46	0.009	0.01	0.29	
