# Supplementary material for: Tree species differ in plant economic spectrum traits in the tropical dry forest of Mexico
Source: PLoS One. 2023 Nov 9;18(11):e0293430. doi: 10.1371/journal.pone.0293430 (PMC10635469; doi:10.1371/journal.pone.0293430)
Supplement: Attachment — Submitted filename: response to reviewers.docx [file pone.0293430.s008.docx]

Reviewer's Responses to Questions

**Comments to the Author**

1. Is the manuscript technically sound, and do the data support the conclusions?
The manuscript must describe a technically sound piece of scientific research with data that supports the conclusions. Experiments must have been conducted rigorously, with appropriate controls, replication, and sample sizes. The conclusions must be drawn appropriately based on the data presented.

Reviewer #1: Partly

Reviewer #2: Partly

We provide all the information requested in methods to support the results and conclusions given

2. Has the statistical analysis been performed appropriately and rigorously?

Reviewer #1: Yes

Reviewer #2: No

We provide more information about statistical analysis in method and results were rewritten to give clarity.

3. Have the authors made all data underlying the findings in their manuscript fully available?
The [PLOS Data policy](http://www.plosone.org/static/policies.action#sharing) requires authors to make all data underlying the findings described in their manuscript fully available without restriction, with rare exception (please refer to the Data Availability Statement in the manuscript PDF file). The data should be provided as part of the manuscript or its supporting information, or deposited to a public repository. For example, in addition to summary statistics, the data points behind means, medians and variance measures should be available. If there are restrictions on publicly sharing data—e.g. participant privacy or use of data from a third party—those must be specified.

Reviewer #1: No

Reviewer #2: Yes

The raw data will be provided as the manuscript is accepted. We added this information in the cover letter.

4. Is the manuscript presented in an intelligible fashion and written in standard English?
PLOS ONE does not copyedit accepted manuscripts, so the language in submitted articles must be clear, correct, and unambiguous. Any typographical or grammatical errors should be corrected at revision, so please note any specific errors here.

Reviewer #1: Yes

Reviewer #2: No

We improve a shorter and clear manuscript. The English of the manuscript was reviewed by AJE experts, if necessary a certify can be uploaded.

5. Review Comments to the Author

Please use the space provided to explain your answers to the questions above. You may also include additional comments for the author, including concerns about dual publication, research ethics, or publication ethics. (Please upload your review as an attachment if it exceeds 20,000 characters)

**Reviewer #1**: Summary
The authors measured woody anatomical traits from 6 species at three sites to examine tradeoffs in wood anatomy and how those may relate to function. The work is generally well presented but I had numerous small comments and concerns mostly around clarity, additional detail, and framing their discussion.

Thanks for your positive comments.

Major comments
-In many cases you make very strong statements about how wood anatomy drives function when you do not have any data on function for your species. You can still discuss the potential links between the two but need to be more careful about discussing your data and the potential links your data have to function based on other published studies.

We appreciate all of your comments that you have made to improve this manuscript. We have read each of your comments and carefully review the text to include all pertinent corrections. We are aware that our discussion is heavy and wordy. We have carefully revised it to reduce its length. We are also aware that there is no direct equivalence between wood anatomical traits and function. Even so, we have been more careful in our statements and we re-write several of them in the discussion. However, all our discussion is supported by other works that, like us, study the same anatomical attributes and reach similar conclusions [*e.g.* 31, 34, 35, 38, 51, 62, 69, 70, 75, 76, 77]; further, although there are fewer of them, there are studies that combine analysis of wood anatomical traits and physiological measurements and we support our discussion on them [*e.g.* 18, 19, 22, 33, 34, 57, 61, 63].

-I am not sure about your modeling approach with AIC. I think it may be a clarification issue, but as I note below in my minor comments, I don’t quite follow the rationale for the approach you took.

We understand your concerns regarding our statistical approach. We have responded to each of your minor comments regarding this and listened to your suggestions and observations. However, we want to make a couple of clarifications in this comment.

1) We do include the interaction of both factors (site and species) as part of our models, we regret this mistake. Now, in table S2 we already include the output of this model. We also carried out a model with only the site as a factor, we mentioned it in our results but we had not included it in table S2 for the simplicity of the table. Now it is also present.
2) although ΔAIC and the likelihood ratio test (LTR) have different approaches, for our purposes either can be used. ΔAIC indicates whether a more complex model is more plausible, while LTR penalizes more complex models that do not explain more variation than they predict. Either way, using AIC or LTR for model selection, both approaches support the same models (see table in S2). The model with the interaction is always the best supported, followed by the model with only the effect of the species. The only difference is that in the model in which the effect of both factors is added, it is penalized by LTR and is not statistically significant. This can be explained because the effect that the site adds by itself explains little variation by itself, so for the TLR adding this effect without interaction penalizes the model and is not significant.
3) Although in our methods we consider a ΔAIC of 2, the value we obtained from the AIC in the comparison of models is greater than 40. So the differences between models are not small.

Minor comments

• Needs to be reviewed for grammar and spelling.

The revised manuscript was edited by a professional company Americna Journal Experts AJE, thus grammar and spelling checked

• Line 66: please note that this is not taking into account vessel length

Yes, we know that vessel length or vessel element length is not considered in the

Hagen–Poiseuille law.

We do not modify the sentence.

• Line 68-69: this is still quite controversial. Please see recent discussion in the literature such as: Isasa, Emilie, et al. "Addressing controversies in the xylem embolism resistance–vessel diameter relationship." New Phytologist (2023). At the very least, you should soften this phrasing to say things like “tend to” or “are often associated with”. Also, clarify that it seems to be species with small vessels that tend to have more resistant xylem (rather than smaller vessels are more resistant).

We modify the text and added this reference.

• Most figures and images are of low resolution. Make sure they are high quality for publication.

Most figures were redone.

• Fig 1. Inset graphs too small to read, missing axis labels. Map needs basic requirements of scale bar, north arrow. More detail in caption (ex. red dots = sites).

This figure was redone. We understand that the Walter-Lieth diagrams are small and not easily distinguished, we modified the image so that they can be seen better. Also, we added the source of the data and details about these diagrams are now readable and also in text.

• Table 1: what is the source of this data? Which years were included?

We agree that this information was missing. Now the source of information is given in the caption and the years that the data covers are in figure 1 in Walter-Lieth diagrams.

• Table 2. Source of wood density data? What about variation?

We have added a column indicating the sources for wood density and phenology data. Here we include only average values. However, in our results and in the discussion section we comment more about their variation.

• Line 142: healthy is a very subjective term. Can you provide more exact criteria? Also, do you have any other information on the sample trees like DBH, height, age, etc. Any indication that variation there matters?

We include more precise information. We did measure the height of each individual, we mentioned it in line 164-166, but for clarity, this is now here. Tree height data is indicated in table 2.

Wood samples from five reproductively active adult trees were collected per species at each site. We avoided trees with visible signs of damage or deformation. We tried to ensure that all the individuals were in similar ranges of height. Additionally, we measured the height of each tree since some studies have shown an allometric relationship between tree height and vessel diameter [23, 24, 25].

• Line 143: what is a “wood slide”?

We meant “a sample”

• Line 144: so you analyze only the last three years of growth? When did you collect the samples?

Previous sentences “The wood samples contained at least three growing seasons delimited by growth rings” was incorrect. It was until permanent sections were reviewed when we notice that at least 3 growth rings were present, but generally each transverse section contained more than three. We removed this sentence and added information, now lines 156-160.

We add the information about when samples were collected. The samples were collected during the dry season of 2022, now in line 161

• Line 151: sounds like dry weight per FRESH volume, right? Please add that clarification.

Yes, it is dry weight per fresh volume. It is now specified, line 166.

• Line 164-169. Not enough detail here to replicate this. How did you select the 50 cells to measure? Started with the outermost cell and just worked your way in from there? How did you select a cell to start with? Also, where were these “fields” placed and how was that decided? What does “in” mean? The whole vessel was inside the field or just part of its cell wall would count?

We understand that details are missing. We have added more details about our method for better replicability

Table 2 lists and describes the wood traits measured for the 80 wood samples. We selected 12 traits to understand the relationship between different wood cell fractions and their relationships with vessel traits, fiber size and wall thickness and wood density between the six tree species of the tropical dry forest. Vessel diameter and frequency are the main descriptors in wood anatomy of indirect water conduction capacity and safety as a redundancy factor [47, 48]. The vulnerability index [49] estimates the degree of xeromorphy of the xylem and is the anatomical counterpart of water potential at 50% conductivity loss [50]. Fiber cell diameter, lumen diameter, and wall thickness are associated with xylem resistance to negative pressures [31, 32, 51]. For vessel diameter, fiber cell diameter, lumen diameter, and wall thickness, we took 50 measurements per sample along the radial axis of the wood from multiple growth rings. We start at one end of the sample, in the outermost part of the wood close to the vascular cambium. From there, we advanced in a radial direction. Once the limit of the sample was reached, we returned to the outermost part to continue scanning the section until reaching 50 vessels. Vessel frequency was calculated by counting all vessels in 10 2.2 mm^2^ cross-sections. Similar to vessel diameter, we started at one end of the sample, in the outermost part of the wood close to the vascular cambium and moving radially. It was ensured that each measurement did not include the area of the previous section. Once all the vessels have been counted, the number of vessels per mm^2^ is expressed [47, 48]. All measurements were performed with an IMAGE-Pro Plus (Media Cybernetics) image analyzer ver. 7.1.

• Table 3 needs some more detail. For example, VF is in relation to all other cells including rays etc.

In response to this comment and as a suggestion from reviewer 2, we have provided more information in the main text (now lines 198-201) instead of placing it on the table 3 now Table 2.

• Line 175: need at least one more sentence that briefly explains this method so readers don’t have to read that paper to get the sense for what you did.

Same answer as the previous comment. New information provided in lines 198-201.

• Line 177: per individual, right? So, using your minimum threshold that suggests you coded at least 81,000 points. 900 * 6 species * 5 individuals * 3 sites. Is that correct? And this was all done manually? If so, was there any checking done by someone else to be sure interpretations of fibers vs parenchyma vs vessels were correct?
• Do you know there are no tracheids in these species? If not, you need to explain how those may impact these codes.

Yes, this was done by individual and manually. Although there was no other person to check it, several checks were made at each measurement to make sure no mistakes were made. On the other hand, since the double staining that was used and the characteristics of the wood of the species, it is difficult to confuse between cell types. For example, parenchyma cells of *C. vitifolium* stains in green and is well distinguished from the red fibers and vessels. For the rest of the species, the difference between highly lignified fibers and the parenchyma is very conspicuous. On the other hand, we are sure that our study species do not have tracheids (neither vasicentric or vascular), because we have radial and tangential sections in which the absence of tracheids was confirmed.

• Figure 2C is helpful but a little messy with overlapping circles, gaps, duplicates, etc. Why not also code each point in panel 2B? Wouldn’t that better help us understand? Oh, just realizing 1A has codes and colors in it. They are impossible to see at this scale and make the whole thing appear messy. Why not just do one nice clean image showing the grid points and the cell assignments?

We have modified this figure for a better visualization of the grid points. We kept panels 2A-B because they show a complete section, and we believe that panel 2B completes the figure. We have modified the titles of this image for a better understanding.

• Line 184-198: glad to see that you accounted for subsampling with your linear mixed model.

Thanks for your comment.

• Line 191. Delta AIC of 2 is pretty small. Often we see 4, 8, or even 10 as the thresholds. Why 2? Also, why using AIC? In this case, you have nested models, right? So you can use likelihood ratio test to compare your three models. In fact, with such a simple model formulation, you can just start with a full model and slowly reduce it down. It seems to me your full model should be a species * site interaction. If the interaction is not significant, then go to species + site. Then, if one of those is not significant, you can remove it or just leave it – it shouldn’t matter with your balanced dataset and you can just report the model summary. If you insist on sticking with AIC, please discuss when there are only small differences in AIC and how that impacts the interpretation of results.

Yes, these are nested models. For this type of model, both the AIC and the likelihood ratio test can be used. In our results, the difference between the AIC values are above 50, so it is not a low value. In S2 table we had omitted the information from the model with interaction. We have added the likelihood ratio test together with the AIC values. All this data is in S2 table. As can be seen in S2 table, both model selection criteria support the same models. We also include the model only the effect of the site.

**S2 Table.** Model comparison through the Akaike Information Criterion (AIC) and the likelihood ratio test (LRT) with Species and Site as a factor. The AIC value is presented and in parentheses the difference between the AIC values. For LTR the loglikelihoods vale is presented and the significance level, <0.0001, ***; 0.001, **; 0.01, *.

| Model | Method | V_D_ | V^mm-2^ | F_D_ | F_Dl_ | F_WT_ |
| --- | --- | --- | --- | --- | --- | --- |
| Species*Site | AIC | 40987 (112) | 4724 (111) | 20681 (117) | 20224 (123) | 6388 (71) |
|  | LRT | 20475^***^ | -2344^***^ | -10322^***^ | -10094^***^ | -3176^***^ |
| Species + Site | AIC | 41034 (65) | 4790 (45) | 20711 (87) | 20245 (102) | 6409 (50) |
|  | LRT | -20511 | -2385 | -10345^**^ | -10112^*^ | -3194 |
| Species | AIC | 41043 (56) | 4792 (43) | 20718 (80) | 20249 (98) | 6407 (52) |
|  | LRT | -20514^***^ | -2388^***^ | -10351^***^ | -10117^***^ | -3195^***^ |
| Site | AIC | 41099 | 4835 | 20797 (1) | 20347 | 6459 |
|  | LRT | -20545 | -2412 | -10394 | -10169 | -3225 |
| Null | AIC | 41099 | 4832 (3) | 20798 | 20347 | 6459 |
|  | LRT | -20547 | -2413 | -10396 | -10171 | -3227 |

• Line 192: normally you only do pairwise of all combinations (species X site in your example) if you test for and find a significant interaction. I see no indication that you considered interactions.

We are very sorry for this confusion. This information was missing in S2 Table. Now, we do include the model with the interaction. We have clarified this in the previous comment and this information is already in the S2 Table.

• Line 201: give us a brief summary what this calculation does and how you calculated it (pooled for a species across sites?). If you did pool, how do we know it is not some form of genotypic variability instead of phenotypic? Phenotypic would really be within a site, right? And even then, they may all be expressing similar phenotypes since they are all experiencing similar environmental conditions.

We appreciate your comment to clarify this point for the reader. Now this is specified in the main text as follows:

To explore trade-offs between wood traits, we calculated Pearson’s correlations for all traits with full data of the species studied. Additionally, to quantify the overall variation in each species, we estimated the relative distance plasticity index (RDPI) for anatomical traits [53]. RDPI is used to calculate the distance in one trait between two individuals of a single species under different conditions. RDPI tests the overall plasticity of a species. In our study, the three sites represent different conditions. RDPI measures plasticity in wood anatomical traits of a single species across the three different rainfall regimes.

RESULTS

• Line 208: table S2 isn’t easy to read. If you stick with AIC, just show the delta AIC here.

We modified Table S2, we decide to maintain both values AIC delta AIC, because without these values, we cannot know which model had the least support and compare between them. We have also included the results of the likelihood ratio test in the S2 Table.

• Fig 3. Expand caption to explain all axes more clearly, remind reader of sample sizes, etc. Seems like you are forcing these groups. Why not just use a continuous axis like wood density across species and use regression?

Now Figure 3 is Figure 4. We have added more information to caption. The analyses were done among species, however showed them here in their group. We do not agree that we are forcing these groups, because they were established based on to two important proxies of functional group classifications: wood density and phenology. Furthermore, as the reviewer mentions, the groups do form according to the continuum of wood density from the lowest wood density in *C. vitifolium* to the highest values in *L. divaricatum* and *H. brasiletto*.

**Fig 4. Differences in wood anatomical traits among species**. Boxplots representing full data of wood anatomical traits for each tree species studied. Different letters indicate significant differences among species, according to multiple pairwise comparisons resulting from mixed models. A. Vessel diameter. B. Vessel frequency. C. Fiber cell diameter. D. Fiber wall thickness. Details of the mixed models can be found in S3 and S5 Tables.

• Fig 3 stats: I understand your approach but because you are not providing a ton of information about each model, it is hard to assess if they were run correctly. They do not align with the visual error bars in these graphs that would suggest fewer differences than you found. Supposedly this is driven by your log scaling and your random effects for individual within site, right? Can you show us more of those data in a way that clarifies this? Perhaps some discussion around how much variation was accounted for by random effects at each level would help?.

Now Figure 3 is Figure 4. We have added more information about models in the methods section. In these diagrams there are not error bars; they represent the quartiles of the boxplots. Even so, it is true that the data between species have an overlap, but this is precisely the effect of the large number of measurements made in each individual and the modeling allows discerning which model fits best. In the results section we have given more information regarding intraspecific variation and we also added S3 table.

• Line 212: they actually are differences among species

Now the analyses and results focus in the species and the species interaction with sites.

• Figure 5. More detail in caption. What is each dot representing? A sample? An individual? Remind readers so they interpret correctly. Why forcing groups when it appears there are differences by species within a group and the groups overlap so much? Define all abbreviations. Letters for sig differences should always start with a at the top or left side of the graph. The labels on panel A axes are a bit subjective. I suggest you remove terms like “storage capacity” and instead just label it what it is: “parenchyma fraction”. In your discussion you can discuss if you think parenchyma fraction is an estimate of storage capacity.

Thanks for the suggestion, this figure was improved. We have modified this figure to standardize the abbreviations. Now, the letters for significant differences are approved. We agree that axes in panel A are somewhat arbitrary so we have modified them.

**Fig 5. Wood cell fraction and wood density variation among species.** A. Ternary plot showing the distribution of tree species according to cell fraction. Each circle represents mean values for each sampled tree, and circle sizes indicate mean vessel diameter. B. Cell fraction distribution in the six tree species from the tropical dry forest. *S. pupurea* is placed separately due to its high proportion of fibers, but these are thin-walled septate fibers that store starch grains. C. Mean wood density in each of the species. For cell fraction and wood density, different letters indicate significant differences among species. Colors in A are similar to those in C and indicate each tree species. Cv, *C. vitifolium;* Sp, *S. purpurea*; Tr, *T. rosea*; Pd, *P. dulce*; Hb, *H. brasiletto*; Ld, *L. divaricatum*.

• Line 261. 30%

It is correct, we have modified this in the main text.

• Line 266 “among”

This is correct, we have modified this in the main text.

• Line 277-279. What trait? I don’t really follow this sentence. How are you assessing “fit” here?

We modified the complete paragraph now in lines 306-314.

• Figure 7 caption. Remind reader what a dot represents. Looks like more dots than individuals here. Is each dot a vessel? If so, how does that impact how you ran your statistics? It actually changes for each row of panels, right? Clarify. Why not a continuous axis like total precip here? Briefly clarify vulnerability index. Why does the order of bars change in each panel? Why does the species change in the middle column? Where are the other species? Overall, I found this figure to be confusing.

We appreciate your comment. Now, Figure 7 is Figure 6 and we modified completely the text. Now in lines 315-321.

• Lines 305-314: So you are only showing the significant ones? This is confusing to me. If showing the non-significant ones takes up too much space, you at least need a clear summary in the main text showing which species had these differences and which did not for all variables tested.

No we were not showing only the significant ones. The whole paragraph was re-written, now lines 331|-335

• Figure 8. Similar comments to all other figures about detail in caption. Order of bars changes.

Figure 8 is now Figure 7, we modify caption adding the information missing to be clear.

**Fig 7. Variation among sites in wood traits from a single measurement.** Boxplots showing values obtained from A, vulnerability index; B, wood density and C-D, the three cell fractions. The color in the boxplot represents each site. Ld, *L. divaricatum*; Hb, *H. brasiletto*, Pd, *P. dulce*; Tr, *T. rosea*; Sp, *S. purpurea*; Cv, *C. vitifolium*. Asterisks indicate the level of significance of the differences between sites. **, <0.01; *, <0.05.

• The results text could be shortened by about 1/3 or more without losing meaning. There is quite a bit of redundancy that is confusing.

The whole section was modified. However, the detailed information provided now for figure captions keep the number of words similar.

• Line 328: Correlations between VI and VF as well as VI and VD are of course going to be strong because VD and VF are used to calculate VI.

Yes, you are correct. However, we do not modify the sentence because if we do not mention it, we will not be able to show Figure 8D.

• Figure 9. I guess this is okay as it is but be aware that these multipanel figures are asking a lot of readers when the x- and y-axes are shifting a lot and do not align across panels.

We agree with this. Figure 9 now is Figure 8. In this figure we tried to make reference to the same variable on the x-axis, for example, from A-C the x-axis is vessels diameter and from G-H it is wood density. We understand the complication and for this reason we have generally decided not to abbreviate any variable in order to make it easier to understand each panel.

• Surprised to see no discussion of intra-species relationships. They clearly differ for many of these interspecies relationships.

Based on the main aims of this study we consider that correlations within the species will not contribute to our discussion.

• Line 340: confusing – is it weak or not significant?

The correlation has a low value of -0.22 and its associated p value is greater than 0.5. This information is found in table S4 so in text we only mention that is no statistically significant.

• Table 4: see my concerns about this index in the methods section.

As mentioned previously for the methods section (see below), we have added more information in the main text. Thus no information was added here.

To explore trade-offs between wood traits, we calculated Pearson’s correlations for all traits with full data of the species studied. Additionally, to quantify the overall variation in each species, we estimated the relative distance plasticity index (RDPI) for anatomical traits [53]. RDPI is used to calculate the distance in one trait between two individuals of a single species under different conditions. RDPI tests the overall plasticity of a species. In our study, the three sites represent different conditions. RDPI measures plasticity in wood anatomical traits of a single species across the three different rainfall regimes.

• Figure 10. Personally, I think that a mixing of figures 10 with figures 4 and/or 6 would be more compelling than this version that is separated. It is okay as it currently is but only the most devoted readers will dive into the details of figures 4 and 6 and I think this figure (10) misses an opportunity to visually depict your species with actual images.

Since the results section was modified. Figure 10 was eliminated. This modificaiton does not affect the comprehension of the text at all.

• 393-394: as opposed to lignified parenchyma?

No, there is no evidence that demonstrates an advantage of the nonlignified parenchyma over the lignified one. Some studies only indicate that the nonlignified parenchyma…

• 394: confusing phrasing around “assigns”

We understand this confusion. We modified the sentence.

For its part, in *S. purpurea* the largest fraction of its wood volume is occupied by fibers, while axial parenchyma is almost absent

• Line 408-409. Careful with your discussion of hydraulic resistance here (and elsewhere) you did not measure it. So you need to soften your phrasing and/or rely on citations of studies that did measure it.

We agree with this comment. We believe that we can delete this sentence without changing the meaning of what is being discussed and no functional statements are made.

• Line 513, which studies?

We rewrote the paragraph, now in lines 469-474.

• Line 516: “potentially offering…”

Done we change the words.

• The discussion is stretching beyond what was measured quite frequently and assuming causation when there is often only correlation or in some cases, the authors don’t even have data. This can be remedied mostly with careful phrasing.

We modified when necessary along the discussion, trying to be careful.

• Line 536: and since there is such a strong desire to draw conclusions about function purely from structure measurements, shouldn’t there also be a recommendation here (or elsewhere) to further our understanding of structure-function relationships for diverse tree species?

We eliminate this part and recommendations for future studies are given in other part of the discussion.

• Line 540-546: these are both long complex sentences that are difficult to follow

We rewrote the sentences. Now lines 496-499.

• 582-583: but you don’t have data on water potential, so can you really say this?

We rewrote the sentence. Now lines 517-519.

• Line 597: this statement is too broad, subjective, and over-generalized.

To avoid these wording, we rewrote the sentence. Now lines 528-530.

• Line 598-600. I don’t think “assumed” is the right approach to this sentence. There is quite a bit of data on how anatomy and density are related and how those drive function

We rewrote the sentence. Now lines 528-530.

• Line 603: “great confidence” is subjective. Stick to your data and compare it to other published data. You don’t have any data on functional performance so instead rely on other studies that do without bringing in subjective terms like “great confidence” unless you are backing them up with data.

We delete this statement.

• Line 604: cite these “other studies”

Done

• Line 604 -605: as written, I don’t think your data support this statement and you don’t have it backed up by a citation right now.

The whole sentence was rewritten indicating that our data suggest…

• Line 607: is “no effect” correct or is it “little to no effect”?

We changed as suggested

• Line 608: again, the term “assigned” seems to imply the tree made a decision about what type of cell to make.

We rewrote the sentence.

• Line 610: stretching too far and too strongly to function. I know what you are trying to argue here but you just don’t have the data to back this up and are speculating about function. You need to soften the phrasing.

Yes, it was speculative, we delete it.

• Supporting information needs more detailed captions and/or footnotes so that readers can interpret all abbreviations and some of the context for each table.

Yes, thanks for your comments. Caption of tables and information was added.

• I didn’t see any mention of raw data being posted to a repository for public access. As the authors know, these data are valuable and time-intensive to collect. I would encourage the authors to consider posting the data associated with this publication.

Yes, they will be post.

Reviewer #2: COMMENTS FOR THE AUTHOR

GENERAL COMMENTS:

The manuscript by Marco et al. examines variation in anatomical functional traits of wood across 6 tropical species along three different rainfall regimes. In this study, the author has conducted a lot of work in the field and laboratory experiments, and compared trait variation across different environmental conditions by using almost the same tree species (5 out of 6 species). Overall, the manuscript is worthy to be published unless substantial and proper revision. But there remains several drawbacks and need to be addressed very carefully. The research questions are clear but the whole manuscript is “heavy” and wordy, data interpretation is not clear and didn’t follow by an very well structured and concise discussion. The author has stated that the tree height has little effect on vessel diameter in this study, but lack data supports. Furthermore, the main hypotheses and predictions in the manuscript are too general or already well-known in the ecological literature (e.g. efficiency vs safety), so the novel contribution of the study is not very clear, need to be further explained.

Thanks for your comments. Now, we expect that the revised manuscript resolved the drawbacks since it was rewritten along the text and it has been edited carefully.

SPECIFIC COMMENTS:
TITLE:
“Wood anatomical traits differ along the economic spectrum” sounds confusion, “Wood anatomical traits” should belong to “plant economic spectrum”.

We think it is an excellent suggestion, we have added this word to the main title.

ABSTRACT:
- line 21,“ cell traits” means “anatomical traits”? if so, use term “anatomical traits” is better. Same as the remaining manuscript.

Thanks for the suggestion, we have reviewed the entire manuscript and use only the term "anatomical traits"

- line 30-32, what do you mean?

We rewrote these part of the abstract lines 35-47.

- line 35, typo, “study” not “sturdy”

Thanks for the annotation, we have corrected this.

INTRODUCTION:
In the introduction section, you mainly emphasize the vessel diameter, but didn’t explain clearly why you want to/why is it necessary to quantify the anatomical traits, why you aim to compare different rainfall regimes, is it important? Why it’s important to examine the trait variation and trade-offs, what’s the ecological significance or what we can understand based on such trait associations?

We modified several paragraphs of the introduction to be more precise.

-Line 57, what do you mean “end of functional spectrum”, can you explain?

We understand that the economic spectrum has two opposite functional profiles. One represented by hardwood species with high wood density, cavitation-resistant xylems, and facultative deciduous trees. We consider at the other extreme the species with low wood density, obligate deciduous and with sensitive xylems. This is why we kept this sentence in the manuscript.

-Line 71-72, further explain the weak relationship between xylem safety and efficiency, why the recent research differ from other studies, these studies are for tropical forest? Which species are they focus? Need to be further explained and compared.

We modify the sentences to clarify the statement. Now lines 78-82.

-Line 74, what are the specific traits and references, not just mention “other anatomical traits are …”-“trait”--- “traits”

We now mentioned which ones in general, lines 84-85.

-Line 91-92, “cover the full economic spectrum” sounds too absolute, what do you mean “full”? May use “wide economic spectrum” instead.

Thanks for the suggestion, we change it.

METHODS:
- Table1, line 105, more caption information should be added.
Check “coefficient of variation is in parentheses”, 1) for max temperature, there is month information in the parentheses; 2) “coefficient of variation” is calculated based on 12 months of a year? 3) do you mean “coefficient of variation” or “standard deviation”. The coefficient of variation (CV) is defined as the ratio of the standard deviation to the mean, and the unit is not “mm”.

We agree that this information is missing and that we had made a mistake. We have clarified what each value in parentheses refers to and added more information in caption. We have moved Table 1 to supporting information and now is in S1 Table.

**S1 Table. General climate characteristics of the study sites.** Mean values; coefficient of variation is in parentheses for annual precipitation and temperature; for rainy season precipitation, accumulated percentage and for the maximum temperature, the month that presents this value is indicated Data was obtained from Clicom Project, National Meteorological Service, CICESE, <http://clicom-mex.cicese.mx>.

- Figure 1, figure resolution is very low, it’s hard to read the text of subplot.

We understand that the Walter-Lieth diagrams are small and not easily distinguished. We have modified the image so that they can be seen better. Also, we added the source of the data and more details about these diagrams are now readable.

**Fig 1. Location of study sites and** **Walter-Liet climate diagram.** Red dots in the map indicate the location of each site. Walter-Liet diagrams were made with data from the nearest meteorological stations at each site (20333-Huatulco for Parque Nacional Huatulco; 17014-Temixco for Sierra de Montenegro; 14019-Autlán for Sierra de Manantlán). In the diagrams, the red dotted area indicates drought months in which the temperature exceeds precipitation. Vertical blue lines indicate the rainy season, and the solid blue area indicates months in which the precipitation exceeds 100 mm. The data were downloaded through the CLICOM system of the Centro de Investigación Científica y de Educación Superior de Ensenada, Baja California (CICESE). The map was made with ArcMap 10.5 [40].

- Line 115-116, how about “June”, rainy season? Now it’ missing.

We appreciate this observation. It was a mistake. Indeed, it should say June to October, we modified it.

- line 125, no unit for 40.9

We have added the units.

- line 131, how do you define “extreme of the economic spectrum” and “intermediate species”, do you compare the traits of multiple tree species in all study sites? More information should be provided earlier, e.g., in INTRODUCTION or somewhere in this METHOD section.

Yes, this paragraph was modified to avoid using intermediate or extremes

Invertir la informacion lineas 137-142l

- Table 2. Wood density data needs to be aligned.

Thanks for the annotation we have corrected this.

- line 129, you selected two species for each site, but how many replicates do you have? I.e., how many individuals for each tree species. Such information should be provided.

This information is provided a few lines below. We selected 5 individuals of each of our six species at the three sites. That is, 5 individuals X 6 species X3 sites=90. However, in two sites we did not find two species. This left us with 80 individuals. All this is specified in this section.

- line 145, one sample was used to calculate...

Thanks for the annotation we have corrected this.

- line 142-151, description of wood density measurement can be shorter and concise.

That is correct, we have modified this section to make the description of the measurement of wood density more concise.

- line 155, use specific alcohol level rather on only “from 50% to 100%”.

We have corrected this.

- line 156-157, what’s the magnification of the lens.

We modified the sentence because different objectives were used depending on the cell type to measure.

- line 157, given the important relationship between tree height and vessel diameter, the information of “tree height” should provided somewhere in the Method section, e.g., provide in Table 2.

Thanks for this suggestion, we add in table 2 the mean ±SD values of tree height for each species.

- line 162, not clear, “80 individuals” mean all tree individuals? Same as line 165, the “individual” shown hear means each thin section for anatomical trait measurement?

As we have clarified in a previous comment, we selected 5 individuals of each of our six species at the three sites. That is, 5 individuals X 6 species X3 sites=90. However, in two sites we did not find two species. This left us with 80 individuals. All this is specified in this section. However, we have changed "individuals" to "wood samples"

- line 163-164, sounds confusion, “their relationships with vessel traits and wood density between functional groups”, why only vessel traits but no fiber wall thickness?

Yes, in fact, we also wanted to compare with fibers wall thickness. We have modified this section and have made it more detailed on why measure these traits.

We selected 12 traits to understand the relationship between different wood cell fractions and their relationships with vessel traits, fiber size and wall thickness and wood density between the six tree species of the tropical dry forest. Vessel diameter and frequency are the main descriptors in wood anatomy of indirect water conduction capacity and safety as a redundancy factor [47, 48]. The vulnerability index [49] estimates the degree of xeromorphy of the xylem and is the anatomical counterpart of water potential at 50% conductivity loss [50]. Fiber cell diameter, lumen diameter, and wall thickness are associated with xylem resistance to negative pressures [31, 32, 51].

- line 167, confusion, why “10 fields”? You didn’t mention it in earlier text.

We understand that details are missing. We have added more details about our method for better replicability.

For vessel diameter, fiber cell diameter, lumen diameter, and wall thickness, we took 50 measurements per sample along the radial axis of the wood from multiple growth rings. We start at one end of the sample, in the outermost part of the wood close to the vascular cambium. From there, we advanced in a radial direction. Once the limit of the sample was reached, we returned to the outermost part to continue scanning the section until reaching 50 vessels. Vessel frequency was calculated by counting all vessels in 10 2.2 mm^2^ cross-sections. Similar to vessel diameter, we started at one end of the sample, in the outermost part of the wood close to the vascular cambium and moving radially. It was ensured that each measurement did not include the area of the previous section.

The reference is given in text. We do not link because it is not a free software.

- line 172, better to use “six tree species”

Thanks for the annotation we have corrected this.

- Table 3, why you choose these 11 traits, what are their main function? Any supporting references? You may provide such information as text or provide in Table 3.

We have provided more information in the main text. Now the new paragraph is in lines 174-191.

- line 176, “each individual” means each thin section?

Yes, we have modified this for a better understanding.

- line 178, add reference or link.

We have added a reference in the main text.

- Fig. 2, nice visualization but the caption is not complete. What do the green dots marked with no.4 refer to?

We have modified this figure for a better visualization of the grid points. We keep panels 2A-B because they show a complete section, and we believe that panel 2B completes the figure. We have modified the titles of this image for a better understanding.

- line 184, two...”models” not “model”

Thanks for the annotation we have corrected this.

- Line 191, model with lowerΔAIC is more plausible, do you mean ΔAIC<2.0 rather than >2.0?

Its correct, we have corrected this.

- line 192, “were” not “was”

Thanks for the annotation we have corrected this.

- line 196-197, ANOVA assumes that the residuals from the ANOVA model follow a normal distribution. When you conduct log-transformation, whether the residuals meet or close to the assumption? Basic plots should be provided in the supplementary (e.g., Q-Q plot, histogram of model residuals) to show that transformed data is better.

We decided not to add this information in supplementary material. If you consider they are important to the understanding of the results we will add them.

- Statistics in Method section not clear.
We have modified all text related to statistics, and we consider these modifications improve the clarity. Now lines 207-233.

RESULTS:

- line 208, ΔAIC > 2.0? “Table in S2, ΔAIC > 2.0 compared to the null model and p<0.05 in pair-wise comparisons” can be simplified into “Table S2, P<0.05”.

We agree with this correction. We have modified the main text.

- line 215, “in the study species”--- “of the studied tree species”

We agree with this correction. We have modified the main text.

- Fig. 4, nice illustration!

Thanks, this figure shows the diversity.

- line 230, what do you mean “supported that the null model”, where is your output of LMMs? I failed to find the corresponding outputs in the main manuscript.

These LMM data is found in the supplementary material in Table S2 and table S3. Additionally, as suggest of the reviewer 1, we have added the likelihood ratio test (LTR) for the model selection. We do include the interaction of both factors (site and species) as part of our models, we regret this mistake, in table S2 we already include the output of this model. We also carried out a model with only the site as a factor, we mentioned it in our results but we had not included it in table S2 for the simplicity of the table. Now it is also present.

- I may delete the line 229-231, and start with “The species of group I had the...”.

We agree with this correction. We have modified the main text.

- line 231, “The species of group..., even differing between the two species” isn’t logically correct, “trait value” should be the subject, e.g., anatomical trait values differed between the two tree species.

We agree with this correction. We have modified the main text.

- for the result section, please provide the mean±SD of the trait value, not use “above 200 µm”, “just above 150 µm”, “above 90”.

We agree with this correction. We have added the mean values and their standard deviation.

- line 252, very confused by the sentence “but not between the species of group II and II.”

We believe that this sentence is repetitive with respect to the rest of the paragraph, so we decided to eliminate it.

- line 253-256, what is the difference between the two sentence!! the first sentence refer to a specific species? Why you use “In contrast”?
“Fiber fraction was always close to 70% in group III, while the parenchyma fraction was rarely greater than 30%. In contrast, the parenchyma fraction of Cochlospermum vitifolium was always close to 70% and fiber fraction was rarely greater than 30%.”

As mentioned, we modify the results and this was rewritten.

- line 267, “Lower case letters in B and C indicate different among species.” not correct. Do you want to say “Different lower case letters in B and C indicate significant differences”?

In figure 5 caption, we agree with this correction and we have also modified the main text.

- line 227, suddenly occurs the “moisture gradient” without any description in Introduction section, why not just use “rainfall gradient” or explain the relationship between moisture and rainfall regimes earlier in Introduction or Method section.

We agree with this correction. We have modified the main text. To avoid confusion of terms we only use "rainfall gradient"

- line 277-279, why? Which figure to support your statement?

The statement that support the results now are in the supplementary. Tables in S3, S4 and S5 show the model selection, output and pair-wise comparisons.

- line 303, Fig. 7, not “Lower case letters indicate differences” but “different lower case letters indicate differences...”

Figure 7 is now Figure 6, we modify not only the caption text but also the graphs.

- line 305-306, please be aware that you are comparing site variation. “we found statiscally (typo, statistically) significant differences only for L. divaricatum” is not correct, should mention “significant difference among three sites”.
- line 307, typo “statistically” not “statically”

Thanks for the comment, we check carefully the spelling.

- line 317, “site” not “Site”, add “different lower case letters indicate differences...” for Fig. 8

Figure 8 now is figure 7 and it was redone.

- line 324, if you say that there is a negative correlation, please add (coefficient ; P-value)

The sentence was modified.

- Fig. 9, it’s obvious that the correlation is mainly driven by groups, how about the trait association within each group, will it differ a lot? Why?

As mentioned for reviewer 1, based on the main aims of this study we consider that correlations within the species will not contribute to our discussion.

- Fig. 10, blue color indicates parenchyma cells while grey color indicates fibre cells? Where is the vessel information shown in figure 10? current version is not complete, figure 10 needs to be further adjusted for better visualization.

This figure was eliminated

- it’s a “heavy” result section, too many figures and tables, maybe remove part of them to supporting material, e.g., Table 1. General site information & Table 3 basic trait information, and some figures.

You are right, it was a heavy result section. Now we modified the text and eliminated one table and two figures

DISCUSSION:

- line 395, “wood volume” rather than “wood”.

Done

- line 420, remove “very” or change to “very thick walls”

Done

- line 448-449, what do you mean “group II fibers are characterized by their very thick walls allowing them to vary parenchyma fraction.” group III also has thickened fibres but why group III has low parenchyma fraction? Is this just your personal defination or there is any causal relationship between thickened fibres and parenchyma?

It was a confusing paragraph and we decide to delete because it does not affect the discussion.

Line 452, avoid redundancy, delete “As mentioned in the results, for”

Done

- line 462-263, why “since”, I didn’t find the causal relationship between “small changes in vessel diameter have important effects” and “the largest variation in vessel diameter and frequency”

The sentences were rewritten.

-line 465-466, mention which three species.

Done

Line 497, change “what was previously thought” to “expected”

Done

-line 511, which two species? Need to be mentioned

Done

- line 513, have you already read all related studies and found the same conclusion that significant variation between fibre and parenchyma? Be careful to use the term “all studies”, which sounds too extreme, maybe you can use “previous studies have shown that [with references]”

We rewrote the sentences to avoid confusion and we added references.

- Line 533-534, I didn’ get it, can you further explain “ there is low variation in these functions and therefore the greatest selection operates on the vessel traits.”

We modify the paragraph and delete part of previous text.

- line 635, should mention which three cell types

We decided not to add the names because along the text they were mentioned.

- it’s good to see that the author discussed the relationship between tree height and vessel diameter and mentioned that there is a weak correlation between tree height and vessel diameter in this study, however, no data was shown to support the statement, it’s necessary to show the height information somewhere in the manuscript and conduct basic statistics to test the effect of tree height on vessel diameter.

Data is mentioned in the main text and table 1. In addition, correlations are presented in the table S7.
